# Molecular Determinants of Per- and Polyfluoroalkyl Substances Binding to Estrogen Receptors

**DOI:** 10.3390/toxics13110903

**Published:** 2025-10-22

**Authors:** Sahith Mada, Samuel Jordan, Joshua Mathew, Coby Loveranes, James Moran, Harrish Ganesh, Sivanesan Dakshanamurthy

**Affiliations:** 1 College of Computer, Mathematical, and Natural Sciences, University of Maryland, College Park, MD 20742, USA; 2 College of Science, Virginia Polytechnic Institute and State University, Blacksburg, VA 24061, USA; 3 College of Arts & Sciences, Georgetown University, Washington, DC 20057, USA; 4 College of Humanities and Sciences, Virginia Commonwealth University, Richmond, VA 22043, USA; 5 Department of Oncology, Lombardi Comprehensive Cancer Center, Georgetown University Medical Center, Washington, DC 20007, USA

**Keywords:** PFAS, QSPR and QSAR modeling, estrogen receptors, molecular docking, hydrophobic interactions, endocrine disruption, binding affinity

## Abstract

Per- and polyfluoroalkyl substances (PFAS) are environmentally persistent organofluorines linked to cancer, organ dysfunction, and other health problems. This study used quantitative structure–property relationship (QSPR) and quantitative structure–activity relationship (QSAR) modeling to examine the binding of PFAS to estrogen receptor alpha (ERα) and beta (ERβ). Molecular docking of 14,591 PFAS compounds was performed, and docking scores were used as a measure of receptor affinity. QSPR models were built for two datasets: the ERα and ERβ top binders (TBs), and a set of commonly exposed (CE) PFAS. These models quantified how chemical descriptors influence binding affinity. Across the models, higher density and electrophilicity indicated positive correlations with affinity, while surface tension indicated negative correlations. Electrostatic descriptors, including HOMO energy and positive Fukui index (F^+^ max), were part of the models but showed inconsistent trends. The CE QSPR models displayed correlations that conflicted with those of the TB models. Following QSPR analysis, 66 QSAR models were developed using a mix of top binders and experimental data. These models achieved strong performance, with R^2^ values averaging 0.95 for training sets and 0.78 for test sets, that indicated reliable predictive ability. To improve generalizability, large-set QSAR models were created for each receptor. After outlier removal, these models reached R^2^ values of 0.68–0.71, which supports their use in screening structurally diverse PFAS. Overall, QSPR and QSAR analyses reveal key chemical features that influence PFAS–ER binding. This predictive approach provides a scalable framework to assess the binding interactions of structurally diverse PFAS to ERs and other nuclear receptors. All the codes, data, and the GUI visualization of the results are freely available at sivaGU/QSPR-QSAR-Molecular-Visualization-Tool.

## 1. Introduction

Per- and polyfluoroalkyl substances (PFAS) are persistent, synthetic chemicals used in cleaning agents, food packaging materials, and industrial processing [1,2]. Structurally, PFAS are a subgroup of organofluorines with strong carbon–fluorine bonds that are highly resistant to thermal, chemical, and biological degradation [3,4]. The high prevalence of PFAS combined with their resistance to degradation has led to increased human exposure. Jian et al. found that concentration levels of perfluorocarboxylic acids in human blood vary between countries [5]. PFAS exposure has been associated with adverse health effects, including cancer, reproductive disorders, and organ dysfunction [6]. Additionally, infants are exposed to PFAS predominantly through breast milk consumption, and their inability to biodegrade PFAS makes them vulnerable to the toxic effects [2]. A possible mechanism to explain the negative effects of PFAS exposure was explained in Kirk et al., who discovered that PFAS can cause the overactivation of nuclear receptors (NRs) [7]. Other studies indicated that the effect of PFAS on NRs such as estrogen receptors (ERs) is not uniform, as PFAS can behave as either agonists or antagonists [8]. These studies emphasize the need to further research how the chemical properties of PFAS impact binding to NRs.

Studies have shown through in vivo experimentation that PFAS bind to ERs with high affinity [9,10]. ER alpha (ERα) and beta (ERβ) regulate gene transcription, which affects reproductive health, brain function, immune response, cardiovascular health, bone integrity, cognition, and behavior [11,12]. The dysregulation of ERs can lead to disturbances in signaling pathways, which results in the onset of various cancers and gynecological disorders [11]. Deroo and Korach found links between ER dysregulation and osteoporosis, neurodegenerative diseases, insulin resistance, lupus erythematosus, and obesity [13]. These studies indicate that PFAS can dysregulate ERs by mimicking their endogenous ligands, which contributes to a variety of adverse health effects.

Prior studies have used quantitative structure–property relationship (QSPR) and quantitative structure–activity relationship (QSAR) modeling to predict toxicity and develop effective drugs. Bhattacharjee et al. developed a 3D QSAR pharmacophore model to predict compounds able to reverse chloroquine resistance [14]. Votano et al. developed QSAR models to predict human serum protein binding using multiple modeling techniques [15]. Studies have used QSAR and QSPR to understand the carcinogenicity of ligands and their effect on lipid accumulation and gene expression [16,17]. Moreover, studies have also used QSAR and QSPR models to predict the binding strength of PFAS to nuclear receptors and calculate the water solubility and vapor pressure of PFAS [18,19]. Therefore, QSAR and QSPR modeling can provide insight into the effect of certain molecular properties in PFAS–ER binding and be used in binding affinity predictions.

While previous studies have developed QSPR models as predictive tools across a variety of ligand structures, this study focuses on creating a targeted approach with two distinct datasets: one for the top PFAS binders and one for commonly exposed (CE) PFAS. The QSPR models were split between the dataset and receptor, creating four unique equations. The purpose of this study is to understand how the molecular properties of PFAS affect their binding affinity to ERs and create QSAR models to accurately predict PFAS–ER binding affinity. This approach addresses the critical gap in understanding how molecular properties of PFAS affect binding strength to ERs while providing a scalable framework for analysis of other NRs.

## 2. Methods and Materials

### 2.1. Receptor Preparation

The orthosteric sites for ERα (1A52) and ERβ (1L2J) were used for docking simulations. These structures were obtained through X-ray crystallography from previous studies [20,21]. The endogenous ligands for each of the receptors were downloaded and separated from their native ERs, while side chains, homodimers, and non-standard amino acids were removed using ChimeraX 1.8, 2024 [22]. Water molecules were deleted, and then hydrogens were added and merged at non-polar protons. Kollman charges were added before converting and exporting the receptors into .pdbqt file types [23]. Grid boxes were created around the orthosteric site for both receptors using AutoDock 4.2 Tools [24]. The grid box size for ERα was 25, 25, 25 (x, y, z), with center coordinates at 11.877, 69.74, 27.89 (x, y, z), and the ERβ gridbox size was 25, 25, 25 (x, y, z), with center coordinates at 106.04, 14.83, 96.83 (x, y, z).

### 2.2. Preparation of PFAS

A total of 14,735 PFAS were obtained from the Environmental Protection Agency’s (EPA) CompTox Dashboard as an Excel file (Table S1) [25]. From this list, PFAS entries without valid SMILES notation were excluded from the dataset. To prepare for docking, Supplementary Codes S1–S3 were used to make a spreadsheet containing SMILES for each ligand, convert these SMILES to .pdb files, and finally produce the .pdbqt files from the .pdb structures. OpenBabel was used to convert from .pdb to .pdbqt, and energy was minimized using the general amber force field [26]. A total of 14,591 PFAS were successfully converted to the .pdbqt file format.

### 2.3. Molecular Docking

AutoDock Vina 1.2.0 software was used to simulate docking and investigate binding interactions [27,28]. The docking protocol and the predictive accuracy of the software were validated through cross-docking experiments with the native ligands of both ERs (Figure S1). Reference ligands had binding conformations within 2.5 Å of their respective computationally docked structures. This confirmed the reliability of the docking simulations in predicting protein–ligand interactions and binding affinities. The complete AutoDock Vina results are presented in Table S2. Additional validation was performed to ensure that the predicted binding affinity values from docking were closely aligned with the experimental results for synthetic ligands bound to ERs. Table 1 and Table 2 present the differences between experimental and AutoDock Vina predicted binding affinities for synthetic ligands targeting ERα and ERβ, respectively. The low standard error, 0.62 for ERα and 0.26 for ERβ, validates the accuracy of AutoDock Vina in predicting the binding affinity of ligands.

### 2.4. Descriptor Selection

During initial descriptor selection for QSPR/QSAR modeling, 30 descriptors were selected from ChemSpider, all of which had descriptor data for each PFAS molecule analyzed [34]. This initial set of 30 was reduced to 15 in order to improve the interpretability and chemical relevance of the final models. Many of the descriptors initially included were either redundant with other variables or lacked clear connections to PFAS–ER binding. For example, all pH-dependent descriptors at pH 5.5 were removed because this pH level is not relevant to human physiological conditions. Eliminating such descriptors focuses the analysis on descriptors that are actually chemically relevant to ER binding.

For the 15 remaining molecular descriptors, the variance inflation factors (VIFs) were calculated for the TB ERβ equation and used to reduce multicollinearity. The descriptor with the highest VIF was removed, and VIFs were recalculated for the new reduced model. This process was repeated until the final reduced model had variables all with a VIF less than 5 [35]. After removing descriptors by VIF, 11 descriptors remained from the original 15. The descriptors removed, in chronological order were as follows: enthalpy of vaporization, logP, polarizability, and boiling point. However, one intentional substitution was made. Although LogP had the second-highest VIF after enthalpy of vaporization, LogP was removed instead of LogD because LogD, at pH 7.4, incorporates ionization effects and is more physiologically relevant to ER binding.

The same set of descriptors for PFAS was used for the other QSAR and QSPR models. This is because PFAS properties such as size, mass, surface area, polarizability, and hydrophobicity are interlinked. These properties tend to correlate between PFAS datasets. For example, average mass correlates with polarizability intrinsically because larger masses correlate with larger molecules, which are more polarizable. Therefore, the use of the same 11 descriptors in each model can be justified by underlying correlations between these physicochemical property descriptors. VIF calculations were run for the same 11 descriptors for the TB ERα model. All VIF values were under 10.

### 2.5. Generation of QSPR Datasets with Descriptors

**Figure 1:**
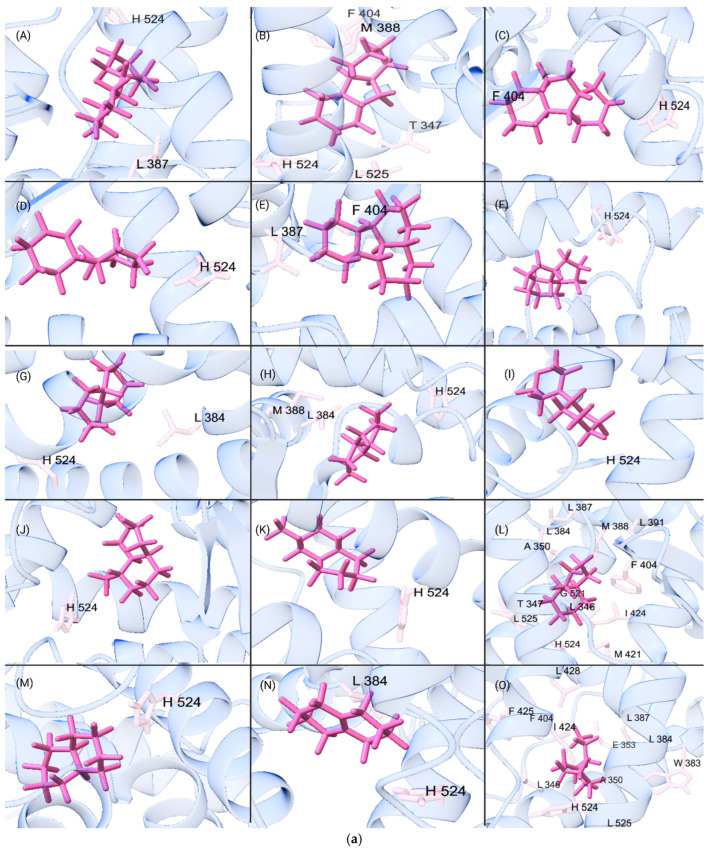

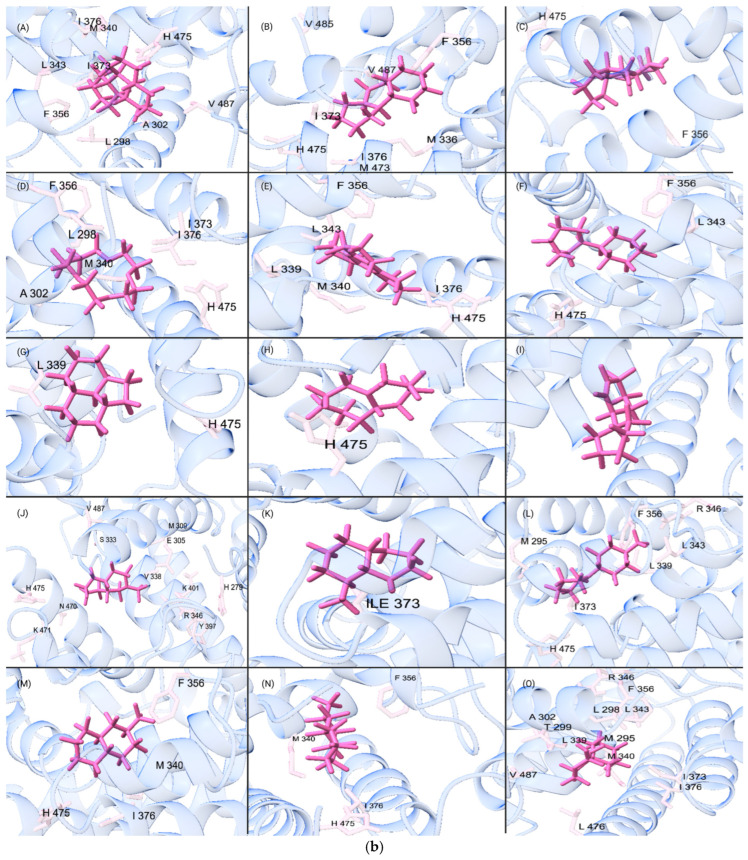
(**a**) Visual representation of the (**A**–**O**) 15 strongest binding PFAS molecules to ERα from the top binders dataset. Key molecular structures and interacting amino acids associated are shown with the ligand represented in purple. (**b**) Visual representation of the (**A**–**O**) 15 strongest binding PFAS molecules to ERβ from the top binders dataset. Key molecular structures and interacting amino acids associated are shown with the ligand represented in purple.

The datasets used in the QSPR analysis for ERα and ERβ were generated from the top 1000 docked PFAS binders. The data was filtered and web scraped, only retaining PFAS molecules with existing ChemSpider data (Code S4). This resulted in 665 PFAS remaining for ERα and 522 PFAS for ERβ. PFAS with the top 69 highest docking scores for both ERs were chosen for further data collection (Tables S3 and S4). Visual representations of the top 15 from the 69 highest PFAS docking scores for ERα and ERβ are shown in Figure 1a and Figure 1b, respectively. PFAS ligands that were missing descriptor data were removed from the datasets (Supplementary Codes S5–S7). A variety of Python packages were used to complete each of these tasks [36,37,38,39,40].

Three PFAS datasets were created for analysis. The first two (Tables S3 and S4) included the top 69 PFAS binders for ERα and Erβ, respectively, with each dataset divided randomly into 55 compounds for training and 14 for testing. These models were subsequently named the ERα and ERβ top binders (TBs) models. The third dataset (Table S5) contained 69 CE PFAS compounds listed in the EPA Toxic Release Inventory with the dataset split randomly into 55 training and 14 test ligands [41]. Chemical descriptors were gathered for all three datasets. Their sources are listed in Table 3, and associated definitions are included in Table S6.

**Figure 2:**
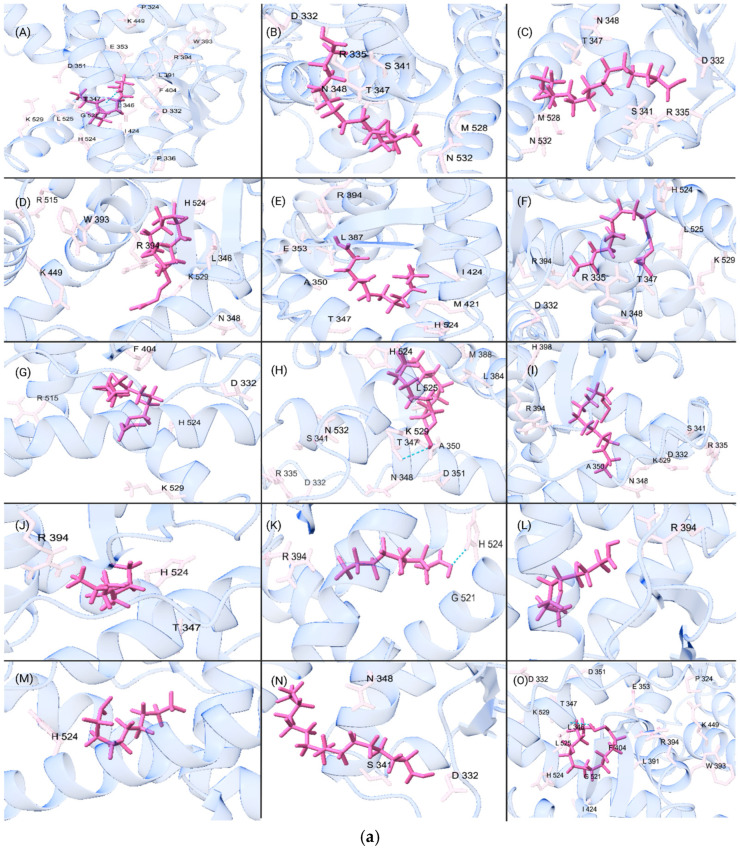

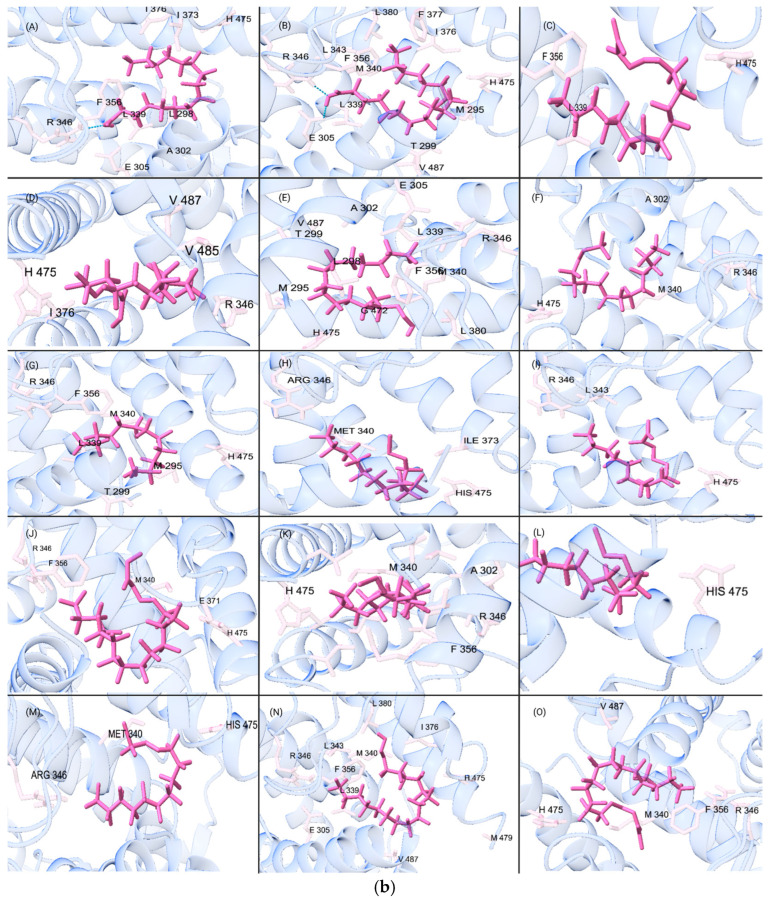
(**a**) Visual representation of the (**A**–**O**) 15 strongest binding PFAS molecules to ERα from the commonly exposed dataset. Key molecular structures and interacting amino acids associated are shown with the ligand represented in purple. (**b**) Visual representation of the (**A**–**O**) 15 strongest binding PFAS molecules to ERβ from the commonly exposed dataset. Key molecular structures and interacting amino acids associated are shown with the ligand represented in purple.

Chemical SMILES strings to calculate the HOMO energy, LUMO energy, and Average Mass descriptors, were input in Avogadro 1.2.0 and geometrically optimized before being converted into MOPAC file extensions [42,43]. The MOPAC extension generated processed output files with LUMO and HOMO energy values and was automated using Codes S8 and S9. Rowan Scientific, Fukui Index workflow (accessed 14 December 2024) was used to calculate Fukui indices and was automated using Code S10 for each PFAS compound. The remaining descriptors were then obtained from ChemSpider [44]. All descriptor values were compiled into Tables S3–S5 for formatting and processing. For the CE dataset, the PFAS and their classifications based on EPA subgroups are shown in Table S7. Figure 2a,b gives visual representations of the top 15 binders selected from the 69 highest CE docking scores for ERα and ERβ.

### 2.6. Development of QSPR/QSAR Model

The QSPR/QSAR models were developed using ChemMaster Basic 1.2 [45] depending on the purpose and training split. The software was used to convert predicted *IC*_50_ (*pIC*_50_) values from the docked *IC*_50_ values via a log-scale format (Equation (1)).

(1) $$pIC_{50}=-{log}_{10}[IC_{50}\;\times \;10^{-9}]$$

QSPR models had a random split assign one ligand to the test set while the remaining ligands were placed in the training set to focus the analysis on the molecular descriptors and their regression coefficients. QSAR models used an 80/20, training/test split to emphasize the predictive capability of the models. The independent variables were set to be the chemical descriptors while the y variable was set to the *pIC*_50_ for each ligand. QSPR equations were generated using multiple linear regressions (MLR).

### 2.7. Applicability Domain Determination

In order to evaluate model reliability and scope, Williams plots were generated for ERα and ERβ using both the TB and CE datasets (Code S11). The plots assessed the prediction error through standardized residuals and structural influence through leverage. Here, an acceptable fit denotes the absolute value of the residual being less than three, while high leverage indicates compounds that lie outside of the model’s structural domain (Equation (2)).

(2) h∗=3(M+1)/N

where M represents the number of descriptors and N represents the number of training compounds [46]. Thus, the Williams plot and these parameters can be used to identify PFAS compounds that lie outside the domain of our models. This potentially identifies additional molecular properties that could increase the generalizability of future binding affinity prediction models.

### 2.8. Assessment of Amino Acid Residue Interactions

The 15 strongest binding compounds from the TB and CE datasets were used to identify the key amino acid residues interacting with PFAS. To determine the frequency of specific amino acid interactions, Code S12 was used to find interacting residues located within a 5 Å proximity of each of the ligands. The 5 Å cutoff is typically used in docking analyses to understand key interactions between ligands and nearby residues within the binding site [47]. The output for each compound was extracted and saved in PDB format, and Code S13 was used to convert the outputs into FASTA format for further analysis.

### 2.9. DUD-E Benchmarks for QSAR Model Validation

The DUD-E benchmark datasets for both ERα and ERβ were used to test the validity of QSAR as a prediction model. This dataset includes known active ligands as well as decoy compounds for ERα and ERβ [48]. It also provides SMILES strings and experimental binding affinities for each compound. Descriptors were collected for all ligands using the same methods described in Section 2.5 (Table S8a,b). After all descriptors were collected, a QSAR model was created using an 80/20 training and test split to predict the binding affinity of each compound. The analysis tested whether QSAR modeling could accurately differentiate between binders and non-binders. This step was used to validate that QSAR modeling is an accurate tool in predicting binding affinity for unknown ligands before implementing it into our workflow.

### 2.10. Development of QSAR Models for QSPR Validation and Affinity Prediction

QSAR models for ERα were developed with 12 experimental binding affinity data values obtained from the literature (Table S9) and added to the TB model of ERα [8,49,50]. A total of 66 models were generated with the methods described in Section 2.6 and each tested on a set of 7 molecules: 5 randomly selected from the TB dataset and 2 experimental compounds. All possible unique pairs of experimental compounds were used across the models. Each training set included the remaining 10 experimental molecules and 45 docked molecules. The molecule from row 41 in the TB dataset was consistently removed to align with previous QSPR analyses, as it was the molecule used in the QSPR test sets. QSAR models were generated with the data found in Table S10. Model performance was assessed using *R*^2^ and *Q*^2^ statistical measurements.

### 2.11. Large-Scale QSAR Prediction Models

To create large-scale QSAR prediction models for the docked PFAS–ER results, the complete docked datasets (Table S2) were used. First, those datasets were filtered with docking score cutoffs of −7.0 kcal/mol and −8.0 kcal/mol for ERa and Erb, respectively. These new datasets (Tables S11 and S12) had molecular descriptors collected with the same methods described in Section 2.5. Then, QSAR models were developed for each dataset using the methods outlined in Section 2.6. In order to account for the standard error of docking, two more refined models were created for each dataset. This was conducted by first removing 10 percent of the highest residuals from the regression, then 20. This outlier removal process preserved the 80/20 training/test split.

### 2.12. Development of GUI for QSAR Molecular Visualization

**Figure 3:**
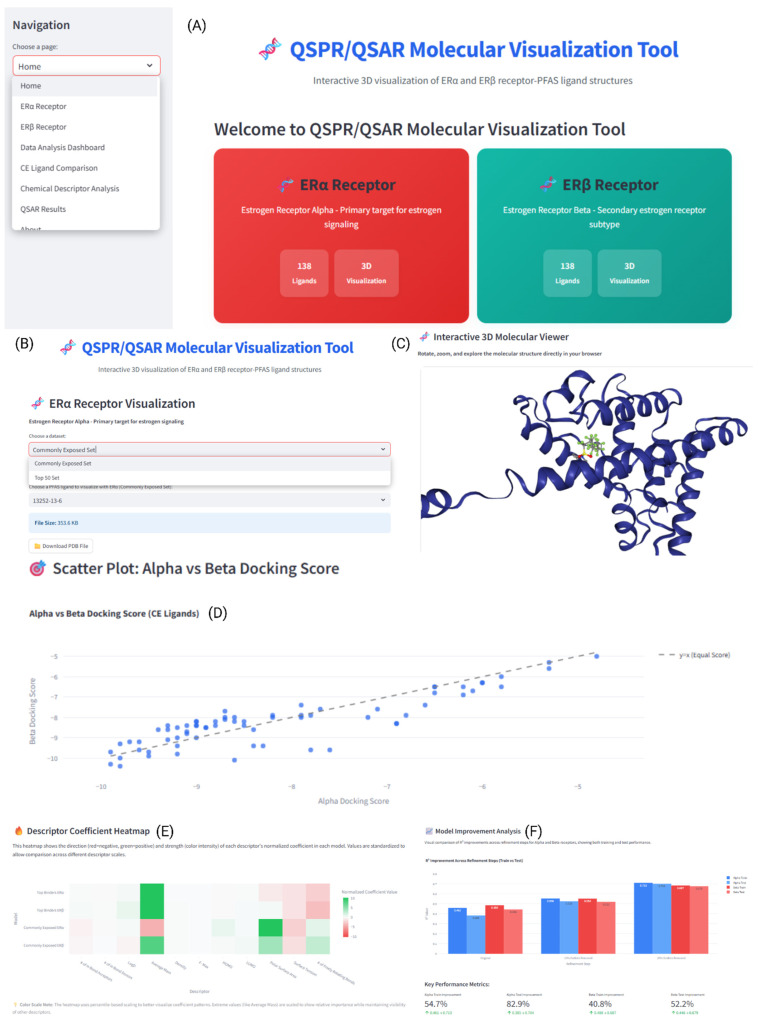
QSAR/QSPR Molecular Visualization Tool. The tool is a custom Streamlit + NGL Viewer web app that packages this study’s results into an interactive visualization. It shows the 276 ligand–receptor complexes (69 CE + 69 TBs for each of ERα and ERβ) in the QSPR models, highlights the bound ligand in the LBD, and supports rotate/zoom/pan for 3D visualization. A left sidebar guides the user to ERα/ERβ structure pages and four analytics views (Data Analysis Dashboard, CE Ligand Comparison, Chemical Descriptor Analysis, QSAR Results). Some of the features include the following: (**A**) Home page that tells the user the purpose of the tool as well as the navigation sidebar. (**B**) Dataset selector (TBs vs. CE) and ligand dropdown, which appear in the live 3D viewer where selected structures can be downloaded. (**C**) 3D molecular viewer with NGL rendering of the selected ER–PFAS complex. (**D**) Alpha vs. Beta docking scatter plot of the CE set to compare Vina scores across receptors to spot PFAS that preferentially bind ERα or ERβ. (**E**) Descriptor coefficient heatmap, which incorporates the normalized QSPR coefficients by model to contrast direction/magnitude of effects across descriptors. (**F**) QSAR model improvement and metrics and bar charts to assess the model improvement in the Large Set QSAR models to see the effect of outlier removal screening.

To display the results of our QSAR study in an accessible format, a Python-based graphical user interface (GUI) named QSAR Molecular Visualization Tool was created (Figure 3).

This application provides an interactive interface for visualizing the PFAS–ER complexes and molecular interactions in real time. The GUI was developed using Streamlit, a Python-based web framework, and NGL Viewer was used for high-quality renderings of the PFAS–ER complexes (Figure 3A). The GUI includes ligand–receptor conformations from both the TB and CE datasets. The molecular visualization component automatically detects and highlights the ligand within the binding pocket. Users can interact with the 3D structures of PFAS–ER complexes through mouse controls: left-click for rotation, scroll wheel for zooming, and right-click drag for panning (Figure 3B,C). A sidebar menu gives direct access to dedicated pages for the 3D structure visualization of the ERα and ERβ complexes as well as four additional analysis visualizations (Data Analysis Dashboard, CE Ligand Comparison, Chemical Descriptor Analysis, and QSAR Results) (Figure 3D–F). The application includes file download capabilities and is freely available for deployment on any system with Python 3.9+ and an internet connection. The web-based tool requires approximately 150 MB of storage and can be downloaded at https://github.com/sivaGU/QSPR-QSAR-Molecular-Visualization-Tool (accessed on 1 October 2025.)

### 2.13. Workflow Overview

**Figure 4:**
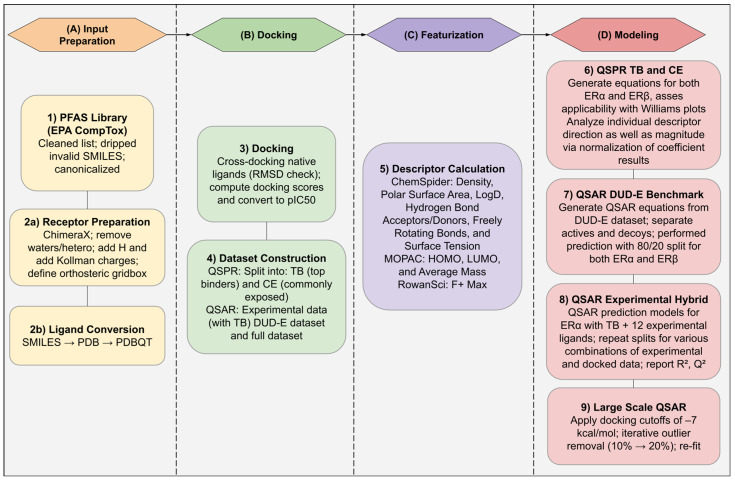
Detailed workflow of the methodology used in this study. It begins with dataset preparation with PFAS curated from EPA CompTox (invalid SMILES removed), ERα/ERβ crystal structures were prepared, and ligands converted to PDBQT then docked with Vina to obtain affinity scores. Two QSPR datasets were built, one top binders (TBs) and another Commonly Exposed (CE) dataset, each with 69 PFAS per receptor. Meanwhile, QSAR models were built using the DUD-E actives/decoys and full docked sets. Descriptors were compiled per ligand from various data sources (ChemSpider: density, PSA, LogD, HBA/HBD, freely rotating bonds, surface tension; MOPAC: HOMO/LUMO, average mass; RowanSci: F^+^ max). QSPR applied multiple linear regression to TBs and CE with applicability-domain checks to relate descriptors to *pIC*_50_. QSAR first benchmarked on DUD-E (80/20 splits) to verify separability of actives vs. decoys, then a hybrid ERα model combined TBs with 12 experimental ligands across 66 unique train/test splits to assess predictive power (reporting R^2^, Q^2^) through assessment of a select set. Finally, large-scale QSAR models on the full docked PFAS sets used docking cutoffs and iterative 10→20% outlier removal to improve generalizability.

The overall workflow of our study is summarized in Figure 4.

## 3. Results and Discussion

The primary goal of this study is to use QSPR modeling to identify and analyze the chemical properties that affect PFAS–ER binding interactions. In addition, QSAR models were used to predict binding affinity of PFAS compounds lacking experimental data. Molecular docking was used to estimate the binding strength of 14,591 PFAS compounds, from which two datasets were derived: one containing the TBs and another containing the CE PFAS compounds. Molecular descriptors related to structure, size, flexibility, and electrostatic properties were collected and incorporated within the QSPR models for each binder (Table S1). The QSPR models were developed using MLR to determine the molecular properties of PFAS that correlate with the predicted binding affinity. QSAR models were then developed as a predictive model and to validate the QSPR findings by adding experimental binding data. First, DUD-E datasets were used to develop QSAR models for both receptors to act as a benchmark validation. Finally, large-scale QSAR prediction models were used to improve the generalizability of binding affinity predictions. The validation table for internal (Train/CV) and external (Test) metric statistics for all models are described in Table 4.

The TB QSPR results indicate that the equation predicts moderately accurate *pIC*_50_ values (Training R^2^ = 0.586 and Test R^2^ = 0.591 for ERα, Training R^2^ = 0.480 and Test R^2^ = 0.582 for ERβ). According to commonly accepted QSAR model criteria, coefficients of determination are generally considered acceptable when R^2^ > 0.6 [51]. Even though the R^2^ values fall slightly below the common 0.60 benchmark, the fitted coefficients are directionally consistent, which allows the correlation analysis of properties on ER binding. The coefficients identify which physicochemical features best correlate with ER binding. While prediction accuracy may be slightly moderate, the models still provide directionality for descriptor-level analysis within the context of the applicability domain. This allows for interpretation of potential links between PFAS structure and ER affinity. The DUD-E benchmark QSAR models all have relatively high coefficients of determination, which surpass the 0.6 threshold in both the training and test sets as well as high internal and external performance metrics. This benchmark is crucial in verifying the predictive accuracy of the QSAR models. This is necessary before applying QSAR to the large-scale PFAS datasets. In the QSAR models for the large-scale datasets, the coefficients of determination are relatively low, which indicates a lack of predictive accuracy. Therefore, outlier screening was performed in order to further refine the dataset, and thus both ERα and ERβ models were able to surpass the 0.6 threshold. This implies the need for large datasets to be refined before application in QSAR models.

### 3.1. QSPR Equations and Molecular Descriptor Coefficients

QSPR equations were generated to quantify the relationship between molecular descriptors and predicted binding affinity. The letter abbreviations associated with the QSPR chemical descriptors are defined in Table 5.

The descriptors were sourced from ChemSpider, MOPAC calculations, and RowanSci, ensuring a consistent and validated dataset across all ligands. Each descriptor is discussed in Section 3.3. The directional coefficients for all the QSPR descriptors in each of the four equations are shown in Table 6.

**Figure 5:**
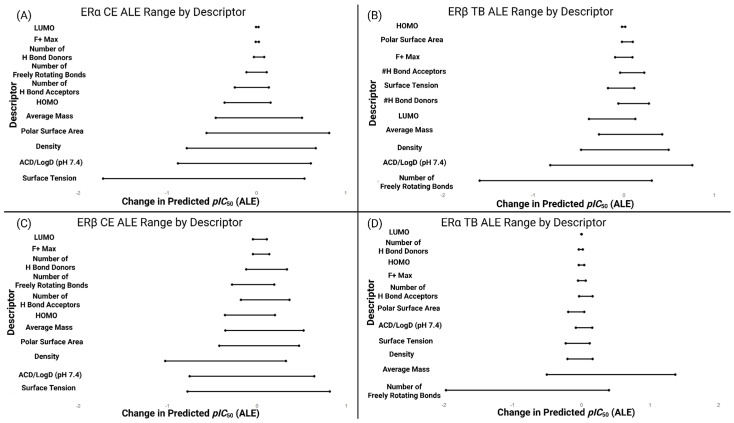
Horizontal segments show the minimum–maximum Δpredicted *pIC*_50_ (ALE) across the empirical range of each descriptor (grid size = 20); endpoints are marked with dots. Shifts to the right of 0 indicate a positive effect on predicted affinity, shifts to the left indicate a negative effect. Panels: (**A**) ERα CE dataset, (**B**) ERβ TB dataset, (**C**) ERβ CE dataset, (**D**) ERα TB dataset. Descriptors include all 11 used in QSPR/QSAR modeling.

In order to contextualize the coefficients in Table 6, model behavior was determined using accumulated local effects (ALEs) for descriptors common to ERα and ERβ for the TB and CE datasets. ALE curves were computed on a 20-point grid and summarized as Δpredicted *pIC*_50_ ranges, capturing the expected change across the observed span of each descriptor while marginalizing over the remaining variables (Code S14). These profiles complement Table 6 by conveying both direction and relative magnitude of impact with reduced sensitivity to multicollinearity (Figure 5).

The patterns align with Table 6: freely rotating bonds and density exhibit the largest magnitude effects in the Top Binder models, whereas PSA, LogD and surface tension dominate in CE, with frontier orbital terms showing comparatively smaller ranges.

**Figure 6:**
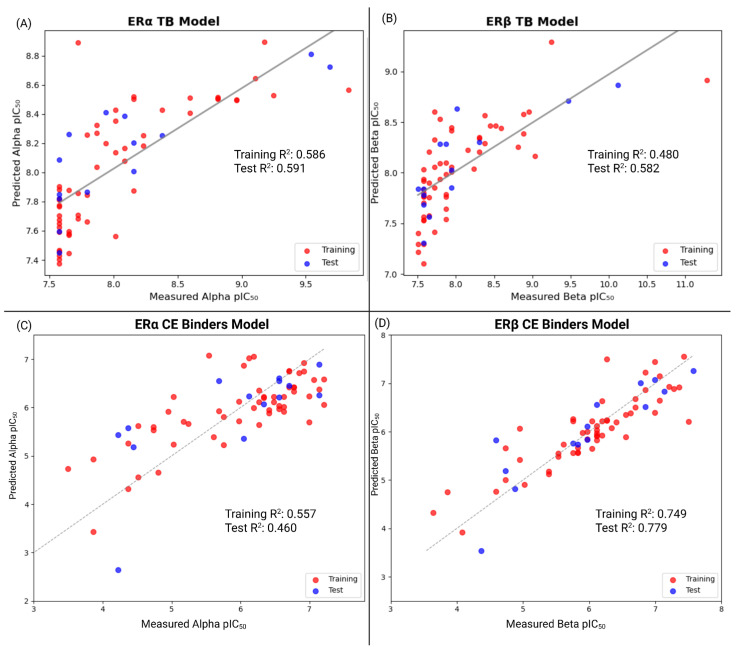
The predicted *pIC*_50_ vs. computationally measured *pIC*_50_ values of the TB and CE QSPR models for both estrogen receptors. The moderate to strong correlations support the relationship between the chosen molecular descriptors and predicted binding affinities across the compounds of each dataset. (**A**) Results of the predicted *pIC*_50_ vs. measured *pIC*_50_ for the TB QSPR model for ERα (Equation (3)). (**B**) Results of the predicted *pIC*_50_ vs. measured *pIC*_50_ for the TB QSPR model for ERβ (Equation (4)). (**C**) Results of the predicted *pIC*_50_ vs. measured *pIC*_50_ for the CE QSPR model for ERα (Equation (5)). (**D**) Results of the predicted *pIC*_50_ vs. measured *pIC*_50_ for the CE QSPR model for ERβ (Equation (6)).

These coefficients capture both the direction and magnitude of each descriptor’s contribution. After incorporating the features into the QSPR equations, the correlation between docked and predicted binding affinities was graphed for both receptors in the TB model (Figure 6).

Overall, the QSPR analysis not only generated predictive equations but also served as a chemical “fingerprint” of the structural and electronic factors affecting PFAS–ER binding. The combination of binding affinity prediction and descriptor analysis in our study serves as a foundation for future QSAR and QSPR research.

### 3.2. Applicability Domain and Williams Plot

**Figure 7:**
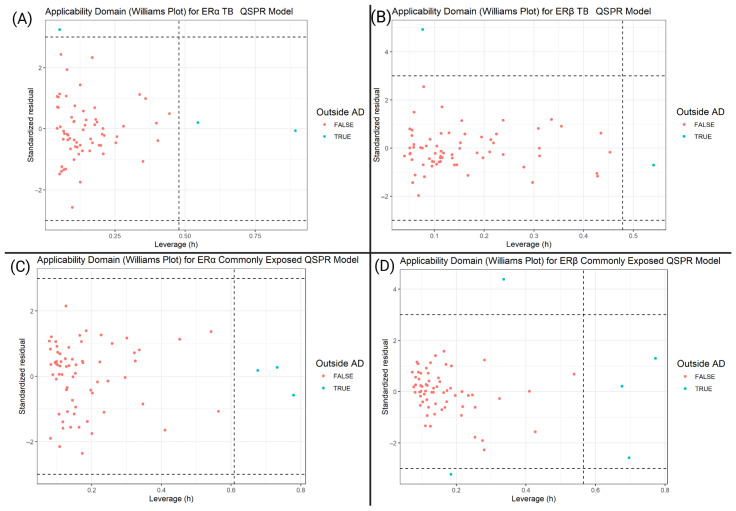
Williams plots (applicability domain) for ERα and ERβ QSPR models. (**A**) ERα TBs; (**B**) ERβ TBs; (**C**) ERα Commonly Exposed (CE); (**D**) ERβ CE. Standardized residuals (*y*-axis) are bounded by horizontal dashed lines at ±3 and leverage ℎ (*x*-axis) is compared to the critical cutoff ℎ* = 3 (M+ 1)/N (vertical dashed line), where M is the number of descriptors and N the size of the training set. Points in red lie within the applicability domain, while teal points fall outside due to excessive residuals and/or leverage.

Figure 7 shows that across all models, the vast majority of ligands fall within the applicability domain, with only a small number exceeding the residual (±3) or leverage (>ℎ*) thresholds.

Figure 6A–D shows that most ligands lie inside ±3 standardized residuals and below the leverage cutoff ℎ*. In the TB models (A, B), the few flagged points are split into two types. The first category is residual outliers with low leverage but large errors, which likely show noise, imperfect linearity, or descriptors that miss key interactions associated with PFAS functional groups. Second are high leverage points with moderate residuals, indicating molecules that are different from the descriptor space in the training set. In the CE models (C, D), nearly all flagged points are leverage-driven with residuals near zero. TB outliers indicate both QSPR/QSAR model fit and coverage issues. This suggests the need for future models to refine the descriptor selection. The CE outliers point to limitations in domain coverage rather than model fit, so future training should include more compounds with similar descriptor profiles to the training set. In practice, predictions beyond ℎ* should be considered low confidence. Overall, the low number of outliers in both leverage and residuals across all four models indicates the reliability of these models in understanding PFAS–ER binding trends.

### 3.3. Analysis of QSPR Results

#### 3.3.1. The Effect of HOMO and LUMO Energies on Binding Affinity

In the QSPR results, electrostatic descriptors such as HOMO and LUMO energies had inconsistent coefficients between models. Within the TB ERα model, the negative HOMO (coefficient j) and positive LUMO (coefficient e) energy coefficients indicate that hydrophobic interactions affect PFAS–ER binding more than electrostatic interactions (Equation (3)).

(3) $${pIC}_{50}=0.22a+0.09d+0.02h-0.02j+0.03e-0.02i\;\;-0.08s-0.38f+0.02g-0.03c+0.05k+8.03\;$$

**Figure 8:**
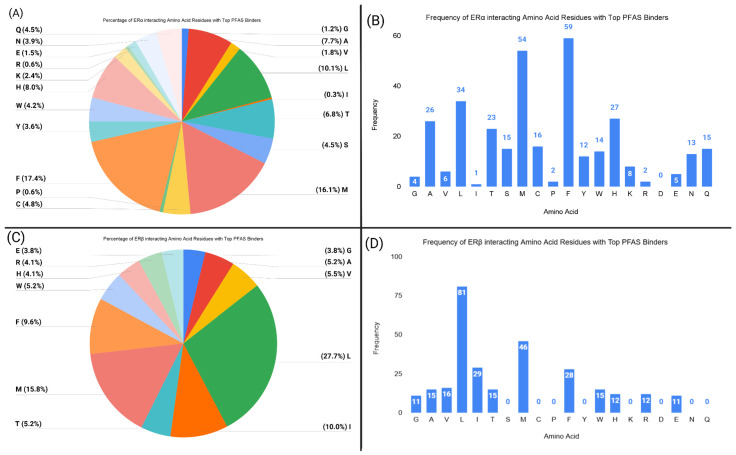
This figure shows the frequency of certain amino acid residues interacting with the top PFAS ligands from the TB models at the orthosteric binding sites of ERα and ERβ. The graphs display the frequency at which each residue interacted with the PFAS ligand within a 5 Å distance. (**A**) Percentage of each interacting residue with the top PFAS binders at the ERα orthosteric site. (**B**) Frequency of each interacting residue with the top PFAS binders at the ERα orthosteric site. (**C**) Percentage of each interacting residue with the top PFAS binders at the ERβ orthosteric site. (**D**) Frequency of each interacting residue with the top PFAS binders at the ERβ orthosteric site.

The coefficients suggest that ligands with higher nucleophilicity or electrophilicity have weaker binding to ERα. In order to explain the results, an amino acid residue analysis was conducted to measure the frequency of receptor residues that interact with the PFAS ligands within a 5 Å cutoff (Figure 8).

PFAS ligands with less electrophilicity or nucleophilicity may form more hydrophobic interactions with ERα. Figure 8 illustrates that phenylalanine, methionine, leucine, alanine, and tryptophan residues can form hydrophobic interactions with PFAS in ERα.

The TB model for ERβ also had positive and negative coefficients for HOMO and LUMO energies, respectively (Equation (4)).

(4) $${pIC}_{50}=0.13a+0.17d-0.05h-0.02j+0.11e+0.01i\;\;-0.06s-0.48f+0.09g+0.04c+0.22k+8.02$$

Despite small amino acid differences between ERα and ERβ, the conserved amino acid sequences between ERs likely explain why both ERs have the same directional relationship with binding affinity for HOMO and LUMO energies. Additionally, the standardized coefficients for ERα and ERβ for HOMO and LUMO energies are similar in magnitude and small, which indicates that those descriptors have a minimal effect on the binding affinity of PFAS to ERs.

The CE ERα model had positive coefficients for HOMO and LUMO energies, respectively (Equation (5)).

(5) $${pIC}_{50}=-0.02a+0.39d-0.05h+0.19j+0.03e-0.48s+0.25f\;\;\;\;\;-0.27g-0.09c+0.42i+0.05k+5.95\;$$

The ERβ model also had positive coefficients for HOMO and LUMO energies, respectively (Equation (6)).

(6) $${pIC}_{50}=0.03a+0.31d+0.03h+0.02j+0.07e-0.29s+0.59f\;\;\;\;\;-0.18g-0.03c+0.15i-0.08k+6.02\;$$

**Figure 9:**
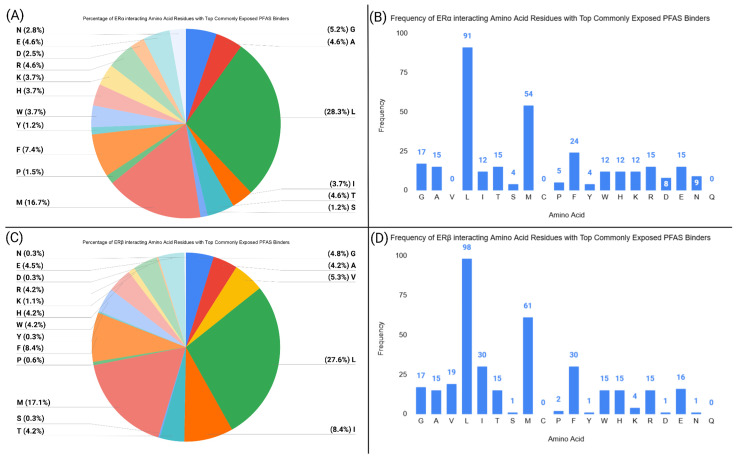
(**A**) Percentage frequency of interacting residues of top commonly exposed PFAS binders at the ERα orthosteric site. (**B**) Frequency of interacting residues of top commonly exposed PFAS binders at the ERα orthosteric site. (**C**) Percentage frequency of interacting residues of top commonly exposed PFAS binders at the ERβ orthosteric site. (**D**) Frequency of interacting residues of top commonly exposed PFAS binders at the ERβ orthosteric site.

The positive HOMO and negative LUMO energy coefficients for ERα suggest that higher nucleophilicity strengthens binding. These coefficients can be explained by the presence of polar functional groups within the CE dataset, which allow for stronger interactions with electrophilic residues of ERs. The flexibility of the CE PFAS allows for these interactions to occur favorably within the LBD of ERs, while the rigidity of the TB ligands prevents these interactions from forming. Furthermore, ERα contains residues that can act as electrophiles for CE PFAS to interact with [32]. For example, the LBD of ERα contains electrophilic residues, such as histidine, arginine, and lysine, which allow for electrostatic interactions with the nucleophilic sites of CE ligands (Figure 9).

Additionally, the magnitude of the CE coefficients is slightly greater than the TB coefficients, which may indicate that HOMO and LUMO energies are more important for determining the binding affinity of PFAS to ERs for the CE PFAS than the TB PFAS. Overall, these results suggest that nucleophilic CE PFAS form stronger interactions with ERs than nucleophilic TB PFAS.

#### 3.3.2. The Effect of Hydrogen Bonding and LogD on Binding Affinity

Hydrogen bond donors (coefficient c) and acceptors (coefficient g) had consistent coefficients between models. In the TB model, the coefficients for the number of hydrogen bond donors were negative and positive for ERα and Erβ, respectively (Equations (3) and (4)). Meanwhile, the coefficients for the number of hydrogen bond acceptors were positive for both ERs (Equations (3) and (4)). Increased hydrogen bond donors and acceptors allow PFAS to form more hydrogen bonds with ERs, with the exception of hydrogen bond donors for ERα. However, the low magnitudes of these descriptors for the TB models (all <0.2) suggest that hydrogen bond acceptors and donors have a mostly positive yet small effect on PFAS–ER binding affinity for top binders.

In the CE model, the number of hydrogen bond donors had consistent negative coefficients for both ERα and ERβ (Equations (5) and (6)). Likewise, the number of hydrogen bond acceptors had negative coefficients for both ERs (Equations (5) and (6)). The negative coefficients for all of these descriptors in the CE models may be explained by the linear structure of the CE ligands. This linear structure allows the CE ligands to form hydrogen bonds with various receptor residues. However, due to the large surface area spanned by these ligands, increased hydrogen bonding ability (donors or acceptors) may cause transient hydrogen bonds that hinder the ligand from maximizing interactions within the LBD. Increased hydrogen bonding ability also correlates with more polar ligands, which may have lower binding affinity to ERs, as most of the interacting amino acids of ERα and ERβ are non-polar (Figure 8 and Figure 9).

LogD (pH 7.4; coefficient k) was a consistent positive predictor of binding strength in the TB model and had positive coefficients for both ERα and ERβ (Equations (3) and (4)). The positive coefficients for LogD indicate that ligands with higher LogD may form stronger hydrophobic interactions with the binding sites. LogD includes both ionized and unionized forms of a ligand at a specific pH. LogD is therefore more accurate to physiological conditions, as it includes ionization states at pH 7.4, and its higher coefficients indicate that hydrophobicity strengthens PFAS–ER interactions. In the CE model, LogD at pH 7.4 had a positive coefficient for ERα and a negative coefficient for ERβ (Equations (5) and (6)). Three out of four models have positive coefficients for LogD, indicating that hydrophobic interactions increase binding affinity. The primary structure differences between ERs may also contribute to these results [12].

#### 3.3.3. Impact of Surface Tension on Binding Affinity

Surface tension is another chemical descriptor associated with IMFs and connected to hydrophobicity. In both the TB and CE models, surface tension (coefficient s) had negative coefficients for ERα and ERβ (Equations (3)–(6)). PFAS with higher surface tension tend to have stronger IMFs and are more polar. Thus, these ligands lack the non-polar sites required to form crucial hydrophobic interactions with ERs [52,53]. The coefficients for surface tension are consistent with EoV, which further suggests that the hydrophobicity of PFAS is necessary for strong binding to ERs.

#### 3.3.4. Impact of Flexibility on Binding Strength

In the TB model, the number of freely rotating bonds had negative coefficients for both ERα and ERβ (Equations (3) and (4)), as lower freely rotating bonds correlated with more cyclical structures. These cyclic structures may mimic the endogenous ligands of ERs and increase binding affinity. For example, the top 10 PFAS binders for both ERα and ERβ all have 1 or less freely rotating bonds and contain cyclic structures. In contrast, the CE model had coefficients for freely rotating bonds that were positive for both ERα and ERβ (Equations (5) and (6)). This positive correlation may be due to the CE dataset containing mostly linear PFAS, which contrasts the polycyclic PFAS found in the TB dataset. Linear CE ligands have more freely rotating bonds, enabling them to fold and form ideal interactions with ERs.

#### 3.3.5. The Effect of Density on Binding Affinity

Density had a consistent positive correlation with binding affinity, explaining why PFAS with more conjugated rings and higher fluorination have higher binding to ERs. In both the TB and CE model, density (coefficient d) was a positive descriptor for both ERα and ERβ (Equations (3)–(6)). These results indicate that higher-density PFAS have less steric hindrance within the LBD of ERα and ERβ than lower-density PFAS. Higher-density ligands may also correlate with cyclic, conjugated structures that are similar to the endogenous ligands of both ERs. Furthermore, higher-density PFAS can form more hydrophobic contacts with the ERs.

In the TB model, the coefficients for average mass (coefficient a) were positive for both ERα and Erβ, respectively (Equations (3) and (4)). Ligand mass correlates with size, and larger ligands can form more interactions with ERs. Furthermore, ERα and ERβ share low amino acid identity in some domains, but the moderate amino acid identity of their LBDs (55%) may explain the positive relationship with average mass and binding affinity for both ERs [54]. Conversely, the CE coefficients for average mass squared were negative and positive for ERα and Erβ, respectively (Equations (5) and (6)). The aforementioned amino acid differences between ERα and ERβ may be more important for the CE dataset due to their structure. These ligands are mostly linear, and larger linear ligands must fold more to fit favorably within the LBD of ERs. However, specific non-conserved amino acids in ERβ may accommodate larger PFAS compared to ERα. Further research into how straight and branched PFAS interact differently with ERα and ERβ is needed to reveal the mechanisms behind these findings.

#### 3.3.6. Binding Affinity Correlations with Fukui Index and Polarity

F^+^ max, the maximum positive Fukui index, quantifies the electrophilicity of the most electrophilic atom in each ligand. The F^+^ max (coefficient h) coefficients were positive and negative for ERα and ERβ in the TB model, respectively. For the CE model, the coefficients for F^+^ max were negative and positive for ERα and ERβ, respectively. The standardized regression coefficients in both the TB and CE models were all very small (all <0.005), and F^+^ max had the lowest magnitude out of all descriptors in each standardized model. This indicates that the descriptor has a very minimal effect on binding affinity and is almost negligible. Although polar and electrostatic interactions (related to F^+^ max) affect the strength of PFAS–ER interactions, our results suggest that F^+^ max is not a good predictor of binding strength in the context of this study.

The polar surface area (PSA) coefficient (coefficient i) for ERα was negative, while for ERβ it was positive in the TB QSPR model (Equations (3) and (4)). Lower PSA correlates with more non-polar ligands, which may increase the hydrophobic interactions crucial for ERα binding [55]. Meanwhile, ERβ better allows for polar regions of ligands to engage in hydrogen bonding and electrostatic interactions with residues in its binding site. In contrast, ERα may favor non-polar contacts due to amino acid differences with ERβ. Conversely, the CE model had positive coefficients for both ERs (Equations (5) and (6)) which may be explained by the presence of amphipathic PFAS compounds in the CE dataset. The non-polar sites of amphipathic PFAS can form hydrophobic interactions that support stronger ER binding. Meanwhile, the polar sites of these PFAS can form hydrogen bonds, electrostatic interactions, and salt bridges with the polar residues of ERs. The higher flexibility of the PFAS ligands in the CE dataset also allows for better positioning of the polar sites to interact with ERs. Overall, the QSPR results give insight into how molecular properties affect PFAS–ER binding strength.

### 3.4. QSAR Prediction Models

#### 3.4.1. QSAR DUD-E Benchmark Validation

**Figure 10:**
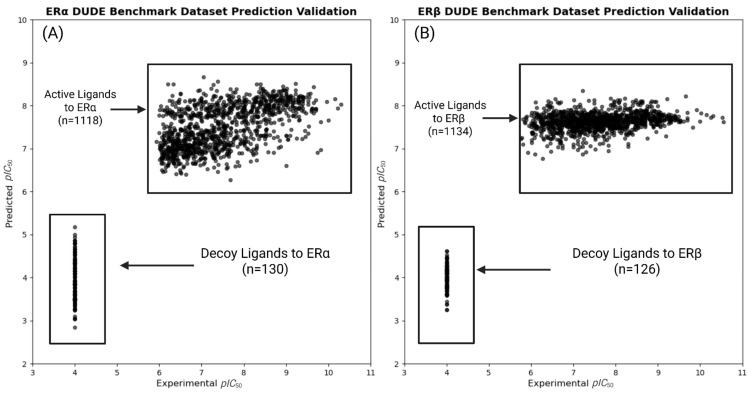
(**A**) The predicted vs. experimental *pIC*_50_ values from QSAR models for ligands binding to ERα. (**B**) The predicted vs. experimental *pIC*_50_ values from QSAR models for ligands binding to ERβ. This figure shows the ability of the QSAR models in differentiating between active and decoy ligands for both ERα and ERβ. Predicted *pIC*_50_ values were generated from QSAR models trained on the DUD-E benchmark dataset combining both active and decoy datasets. Each point in the plot represents a ligand, either active (known binders) or decoy (non-binders), with the model demonstrating the ability of the model to accurately differentiate between either set.

The development of QSAR models with experimental results serves to validate the QSPR results and creates binding affinity predictions. The QSPR modeling predicted the directional effects of molecular descriptors on binding affinity. In order to transition from property relationships to create binding affinity predictions, QSAR models were developed. The QSAR framework was first verified with DUD-E then developed with experimental binders. DUD-E benchmark datasets were used to validate the predictive accuracy of the ChemMaster software and the molecular descriptors used in our QSAR approach. DUD-E is a benchmark virtual screening dataset containing our ER targets (ESR1 and ESR2). Each dataset contains both known active and decoy ligands, which are used to test molecular docking and scoring methods. Descriptors were gathered for both active and decoy ligands using the same procedures outlined in Section 2.5, and the dataset was split randomly into an 80/20 training/testing split. Decoy binding affinities were set to 100,000 nM for standardization as a baseline, as the DUD-E dataset had listed binding affinities of “>100,000 nM” for all decoys. Compounds with KI values listed were converted to *IC*_50_, then *pIC*_50_. Plots were generated to compare the predicted *pIC*_50_ versus experimental *pIC*_50_ values (Figure 10). Explicit thresholds were not applied in this analysis.

The benchmark models demonstrated the ability to accurately differentiate between active and decoy ligands for ERα and ERβ. The minimum difference in predicted binding affinity (*pIC*_50_) between the active and decoy compounds was ~0.93 and ~2.21 for ERα and Erβ, respectively. This corresponds to the weakest predicted active compound having an *IC*_50_ approximately 9-fold and 161-fold lower than the strongest predicted decoy for ERα and Erβ, respectively. These results demonstrate that the selected descriptors are accurate predictors of binding affinity and validate the use of ChemMaster QSAR prediction models in our study.

#### 3.4.2. QSAR Analysis for TB PFAS Combined with Experimental PFAS Affinities

QSAR prediction models were trained on the TB ERα ligand set and 12 ligands with experimentally determined ERα binding affinities. The experimental data was obtained from the literature and was used to generate 66 unique QSAR models with the data split among training and test sets [46,47,48]. These models can predict the binding affinity for structurally diverse PFAS and compounds without experimental or computational binding affinities. The QSAR models provide a reliable method for screening PFAS and assessing their toxicity.

After all the models were run, the average coefficient of determination (R^2^) for the training sets was approximately 0.95, indicating a strong correlation between the selected molecular descriptors (Table 3) and binding strength of PFAS to ERα. The average R^2^ from the 66 test sets was 0.78, which was lower than that of the training set. The high R^2^ averaged across the test sets shows the effectiveness of the selected molecular descriptors in accurately predicting the binding affinity of diverse PFAS structures to ERs outside the initial training set. The models had a high average cross-validated Q^2^ value of approximately 0.86, indicating a strong predictive capability of the QSAR models. The collective statistical metrics (R^2^ training ~ 0.95, R^2^ test ~ 0.78, Q^2^ ~ 0.86) support the use of these QSAR models for potential screening and risk assessment of PFAS compounds. These results validate our computational approach and improve its applicability in predicting ER disrupting effects of PFAS. Future research may validate these findings experimentally and add to a potential regulatory evaluation of PFAS.

#### 3.4.3. QSAR Binding Prediction for Large-Scale PFAS Molecules

**Figure 11:**
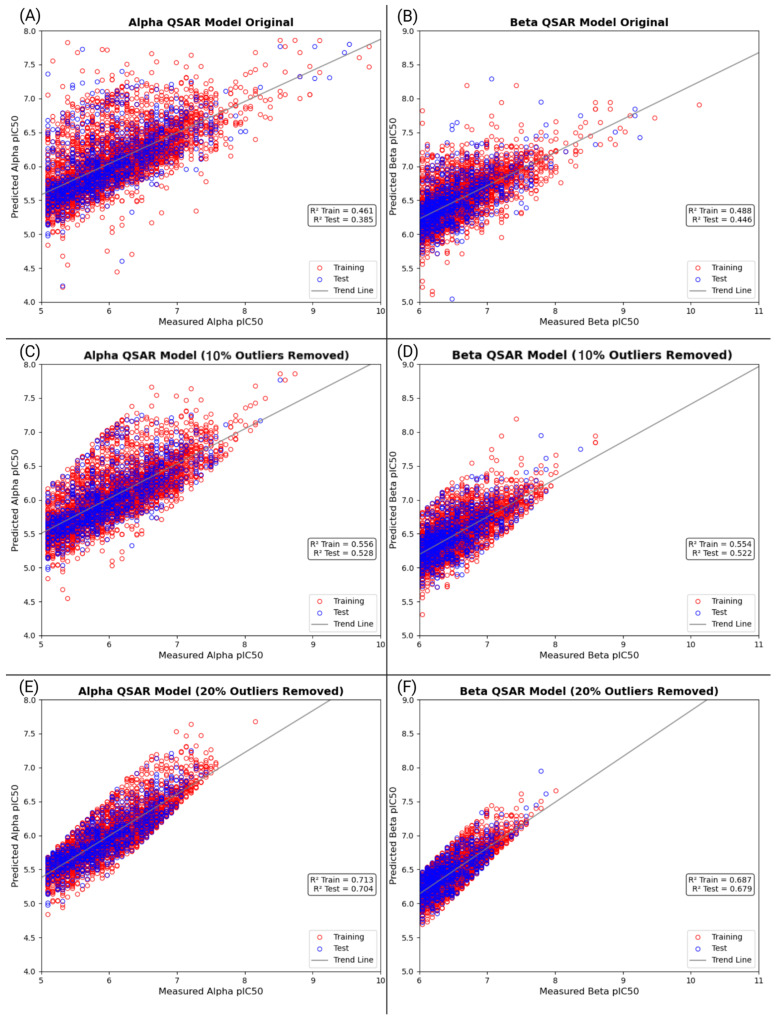
Predicted versus measured *pIC*_50_ values from QSAR models trained on two rounds of dataset refinement for ERα and ERβ. (**A**,**B**) show the models trained on the full selected dataset of docked PFAS compounds for ERα and ERβ without outlier exclusion. (**C**,**D**) represent the first round of refinement, where 10% of the outliers were removed from the full dataset graphs for both ERα and ERβ. (**E**,**F**) display the second round of refinement, where 20% of the outliers were removed from the full dataset graphs for ERα and ERβ. Each refinement step resulted in a stronger correlation between predicted and measured *pIC*_50_ values, indicating that removing outliers improves model performance and may help reduce the noise in the data. These findings support the importance of refinement in building accurate QSAR models when experimental data may not exist.

Prior QSAR models in this study have used smaller PFAS datasets of the top binders and known experimental ligands. This section builds on that framework with larger sets of docked PFAS to improve prediction accuracy and account for the uncertainty in limited sets. The training of QSAR models solely on top binders may reduce the generalizability of the results to diverse chemical structures. Minor orientation changes that occur during computational docking can lead to large changes in predicted binding affinity. This may cause errors between computationally determined and experimentally determined binding affinities. To address these limitations, large-scale QSAR models were developed for both ERα and ERβ from a set of 4895 and 4974 ligands, respectively. Each model went through two rounds of removing outliers to improve predictive accuracy. In the first round, 10% of ligands with the highest residuals were removed for both receptors. In the second round, an additional 20% were removed. In the first round of outlier removal, the coefficient of determination (R^2^) increased from 0.451 (A) and 0.493 (B) to 0.625 (C) and 0.638 (D) for ERα and Erβ, respectively. The second round of removal raised the R^2^ to 0.660 (E) and 0.682 (F) for ERα and Erβ, respectively. The six QSAR models for ERα and ERβ are represented in a graph, with the training sets described by red points and the test sets by green points (Figure 11).

These results indicate that, after removing outliers, the large-scale models effectively represent the relationship between molecular descriptors and predicted binding affinities to ERs. Importantly, the removal of outliers in the models matches the noise reduction approach used in a study [56]. It also is a similar approach to the Monte Carlo cross-validation and outlier removal strategy described by Cao et al. [57]. Overall, the results and modeling workflow for this study are not limited to the ERs, as the framework can be expanded to other NRs. Applying this framework to other NRs will allow for prediction of ligand–receptor binding affinity, improving in silico screening for PFAS and other endocrine-disrupting chemicals.

## 4. Strengths and Limitations

A key strength of this study is the detailed QSPR analysis on a selected subset of ligands from an initial dataset of over 14,000 docked PFAS compounds. Binding affinity predictions were made using AutoDock Vina, where reference ligands showed conformational alignments within 2.5 Å of their experimental crystal structure, strengthening the reliability of the docking results. The broad set of molecular descriptors (Table S2) gives insight into how the structural features of PFAS affect binding affinity to ERs. By analyzing strong binders and common environmental PFAS, our study improves the specificity of the QSPR models. Further in vitro studies are necessary to validate these findings since binding affinity values were computationally calculated. Nonetheless, a key strength of the QSAR models lies in their ability to predict the binding affinity of PFAS compounds not yet experimentally tested. The use of experimental binding data validates our QSAR models, improving generalizability to physiological conditions. However, the lack of statistical significance measures in the ChemMaster Basic 1.2 software prevents the standardization of results and an assessment of whether the findings occurred by chance.

## 5. Future Directions

By identifying key molecular properties that affect PFAS–ER binding, this study advances the understanding of PFAS toxicity and contributes to improved risk assessment strategies. The findings provide a foundation for regulatory policies aimed at reducing the health risks associated with PFAS exposure. Further studies should focus on in vitro and in vivo testing, refinement of computational models, and research of allosteric ER modulation. Additionally, integrating machine learning approaches may further improve predictive accuracy. Expanding beyond traditional QSAR modeling is essential, as it only uses static molecular descriptors, omitting the dynamic interactions that occur during ligand–protein binding. This limits the ability to fully understand the complex factors that affect ligand–receptor interactions. Incorporating advanced computational techniques, such as molecular dynamics simulations, can account for conformational changes in the receptor during ligand binding. Implementing deep learning algorithms, such as neural networks, can improve model performance by detecting non-linear relationships.

## 6. Conclusions

In this study, thousands of PFAS were docked to ERα and ERβ to build QSPR equations for the TB and CE binders. Subsequently, both small-set and large-scale QSAR models were trained to translate these property trends into binding affinity predictions. These models can screen untested PFAS and identify the chemical features most responsible for ER binding. The QSPR results revealed molecular properties with consistent directional effects on binding affinity. Density had a consistent positive correlation with binding affinity, which indicates that higher-density PFAS form stronger interactions with ERs. In contrast, lower surface tension correlated with stronger binding to ERs, indicating that PFAS hydrophobicity plays a key role in strong ER binding. Comparative analyses between the strongest-binding PFAS and environmentally prevalent PFAS show inconsistent correlations between chemical properties and binding affinity. While these inconsistencies do not necessarily mean that different PFAS–ER interactions occur in each dataset, they suggest that environmental PFAS may interact with ERs via different mechanisms that can be investigated in future studies. These differences may occur because the CE ligands contain a variety of functional groups and retain a linear structure, whereas the TB ligands contain more cyclic structures with fewer functional groups. Across models, hydrophobicity-related terms (density, LogD) increased predicted affinity for both receptors, whereas higher surface tension decreased it. In the TB equations, ERα favored compact, cyclic chemotypes (negative freely rotating bond and PSA coefficients), while ERβ favored greater polarity (positive PSA) with a similar penalty for flexibility. In the CE equations, both receptors shifted to favor flexibility (positive freely rotating bond coefficients) with smaller and less consistent contributions from frontier-orbital descriptors. Applicability-domain diagnostics were similar overall, though leverage-driven outliers were more common in CE models, implying greater structural diversity. Collectively, these patterns indicate that ERα binding is driven more by non-polar packing, whereas ERβ accommodates more amphipathic and flexible structures. Additional studies are necessary to validate the mechanisms behind these reasons and challenge or verify these results. These findings provide valuable insights for risk assessment and the future design of safer chemicals with lower endocrine-disruption effects.

## Figures and Tables

**Table 1:** Docking scores and binding affinities of synthetic ligands to ERα with experimentally derived *IC*_50_ values, AutoDock Vina scores, and predicted *IC*_50_ values from AutoDock, along with the differences between AutoDock predicted and the literature [12,29,30] *pIC*_50_ values for each ligand.

Estrogen Receptor Ligands	PubChem CID	*IC*_50_ Values (nM)	*pIC*_50_ Values	AutoDock Vina Score (kcal/mol)	AutoDock *IC*_50_ Values (nM)	AutoDock *pIC*_50_ Values	AutoDock *pIC*_50_ Literature *pIC*_50_ Difference
Hydroxytamoxifen	449459	40	7.40	−9	278.38	6.60	−0.844
Caffeic Acid	689043	120,000	3.92	−6.4	21,800	4.66	0.74
Bazedoxifene	154257	26	7.59	−8.5	643.90	6.19	−1.39
Lasofoxifene	216416	1.08	8.97	−8.4	761.48	6.19	−2.85
Fulvestrant	104741	4.4	8.36	−8.3	900.52	6.05	−2.31

**Table 2:** Docking scores and binding affinities of synthetic ligands to ERβ with experimentally derived *IC*_50_ values, AutoDock Vina scores, and predicted *IC*_50_ values from AutoDock, along with the differences between AutoDock predicted and the literature [12,31,32,33] *pIC*_50_ values for each ligand.

Estrogen Receptor Ligands	PubChem CID	*IC*_50_ Values (nM)	*pIC*_50_ Values	AutoDock Vina Score (kcal/mol)	AutoDock *IC*_50_ Values (nM)	AutoDock *pIC*_50_ Values	AutoDock *pIC*_50_-Literature *pIC*_50_ Difference
Estradiol	5757	46	7.34	−10	52.0	7.28	−0.0535
Raloxifene	5035	7.7	8.11	−11.1	8.79	8.06	−0.0575
QYA	145949437	5000	5.30	−6.97	127,000	3.90	−1.40
Benzoxazole	9228	5.4	8.27	−11.3	6.18	8.21	−0.0586
Hydroxytamoxifen	449459	23,000	4.64	−6.23	36,700	4.44	−0.23

**Table 3:** Data source for each descriptor in the QSPR models.

ChemSpider	Avogadro and MOPAC	RowanSci
Density, Number of Hydrogen Bond Acceptors and Donors, Number of Freely Rotating Bonds, LogD (pH 7.4), Polar Surface Area, and Surface Tension	HOMO, LUMO, Average Mass	F^+^ Max

**Table 4:** Internal and external test statistics for all QSPR/QSAR models for both receptors. Internal performance is reported as training set fit (R^2^, RMSE, MAE) and cross-validated predictivity (Q). External performance is computed on a held-out test set not used for model building. RMSE/MAE are in *pIC*_50_ units, and all QSPR/QSAR models used an 80/20 training/test split.

Model	R^2^ (Train)	RMSE (Train)	MAE (Train)	Q^2^ (Internal)	R^2^ (Test)	RMSE (Test)	MAE (Test)
TBs (ERα)	0.586	0.351	0.253	0.307	0.591	0.432	0.332
TBs (ERβ)	0.480	0.459	0.295	0.191	0.582	0.485	0.356
Commonly Exposed (ERα)	0.557	0.639	0.514	0.139	0.460	0.769	0.604
Commonly Exposed (ERβ)	0.749	0.435	0.315	0.456	0.779	0.465	0.332
DUD-E (ERα)	0.678	0.795	0.648	0.672	0.672	0.867	0.700
DUD-E (ERβ)	0.618	0.845	0.678	0.611	0.672	0.823	0.662
Large-Scale QSAR (ERα)	0.461	0.506	0.388	0.455	0.385	0.525	0.387
Large-Scale QSAR (ERβ)	0.488	0.364	0.274	0.484	0.446	0.364	0.274

**Table 5:** The abbreviations of the chemical descriptors used in the QSPR equations.

a = Average Mass	c = Number of H-Bond Donors	d = Density
e = LUMO	f = Number of Freely Rotating Bonds	g = Number of H-Bond Acceptors
h = F^+^ Max	i = Polar Surface Area	j = HOMO
k = LogD (pH 7.4)	s = Surface Tension	

**Table 6:** Each QSPR model with respective coefficients for each descriptor with green cells representing positive coefficients and red cells representing negative coefficients. (A) Represents the raw coefficients, which have been unstandardized. (B) Represents the normalized coefficients, which have been calculated from the standard deviations of the descriptor data.

**(A) Descriptor Name**	**Top Binders ERα Coefficients**	**Top Binders ERβ Coefficients**	**Commonly Exposed ERα Coefficients**	**Commonly Exposed ERβ Coefficients**
Number of H-Bond Acceptors	0.0154	0.0857	−0.2744	−0.1780
Number of H-Bond Donors	−0.0312	0.0395	−0.0874	−0.0343
LogD	0.0485	0.2245	0.0483	−0.0835
Average Mass	0.2222	0.1336	−0.0198	0.0344
Density	0.0887	0.1731	0.3900	0.3057
F^+^ Max	0.0166	−0.0471	−0.0485	0.0343
HOMO	−0.0198	−0.0218	0.1857	0.0242
LUMO	0.0285	0.1132	0.0316	0.0734
Polar Surface Area	−0.0185	0.0069	0.4216	0.1504
Surface Tension	−0.0821	−0.0635	−0.4753	−0.2855
Number of Freely Rotating Bonds	−0.3843	−0.4810	0.2519	0.5858
**(B) Descriptor Name**	**Top Binders ERα** **Coefficients**	**Top Binders ERβ Coefficients**	**Commonly Exposed ERα Coefficients**	**Commonly Exposed ERβ Coefficients**
Number of H-Bond Acceptors	0.0408	0.1842	−0.4964	−0.3563
Number of H-Bond Donors	−0.0322	0.0363	−0.0487	−0.0212
LogD	0.2422	0.8252	0.1923	−0.3679
Average Mass	49.2514	30.1649	−4.1092	7.8995
Density	0.0219	0.0420	0.0513	0.0445
F^+^ Max	0.0011	−0.0048	−0.0043	0.0034
HOMO	−0.0449	−0.0459	0.7999	0.1153
LUMO	0.0434	0.1306	0.1539	0.3954
Polar Surface Area	−0.9347	0.2707	10.6211	4.1924
Surface Tension	−1.3549	−1.3352	−2.3411	−1.5560
Number of Freely Rotating Bonds	−2.3991	−3.3561	1.0370	2.6684

## Data Availability

All the data generated in this study is presented in the main manuscript and additional data available in the Supplementary Materials and in GitHub. The QSPR/QSAR Molecular Visualization GUI Tool is available on Github at: https://github.com/sivaGU/QSPR-QSAR-Molecular-Visualization-Tool, accessed on 1 October 2025.

## Supplementary Materials

The following supporting information can be downloaded at: https://www.mdpi.com/article/10.3390/toxics13110903/s1. All supporting figures, tables, and code used in this study are available here: Code S1: ChemSpider SMILES extraction from input sheets for canonical SMILES. Code S2: SMILES-to-3D conformer generation; outputs per-ligand PDB files. Code S3: Batch conversion of ligand PDB to PDBQT for docking. Code S4: Integration of top-binder results with identifiers; appends CASRN, name, SMILES, docking score, and ChemSpider references. Code S5: Descriptor scraping for top binders. Code S6: Link seeding for the Commonly Exposed (CE) set; maps CASRN to ChemSpider entries. Code S7: Descriptor scraping for the CE set using the same normalized parsing as Code S5. Code S8: Avogadro-driven automation to generate MOPAC inputs/outputs for HOMO/LUMO and molecular weight. Code S9: Parser for MOPAC outputs; compiles HOMO, LUMO, and molecular weight. Code S10: Batch computation of Fukui(+) with F^+^ max per molecule. Code S11 (R): Williams plot generation for applicability domain; computes leverage, standardized residuals, ℎ* cutoff, flags outliers, and exports figures/tables for ERα/ERβ (TB and CE). Code S12: Automated extraction of receptor FASTA sequences from bound PDB complexes for residue analyses. Code S13: ChimeraX workflow to select residues within 5Å of ligands, label, and export interacting residues for frequency analyses. Code S14 (R): Accumulated Local Effects (ALE) across all 11 descriptors; summarizes effect ranges for ERα/ERβ models to support coefficient interpretation. Table S1: Master PFAS list curated from EPA CompTox used for docking and preprocessing. Table S2: Complete AutoDock Vina outputs for ERα and ERβ with docking scores for all processed PFAS. Table S3: ERα top binders (69 ligands) with identifiers and final descriptor inputs for QSPR modeling. Table S4: ERβ top binders (69 ligands) with identifiers and final descriptor inputs for QSPR modeling. Table S5: CE PFAS (69 ligands) with identifiers and final descriptor inputs for QSPR modeling. Table S6: Descriptor definitions, units, and sources for the 11-descriptor panel. Table S7: CE PFAS classifications by EPA subgroup for stratified analyses. Table S8a,b: DUD-E benchmark datasets for ERα/ERβ with descriptor inputs. Table S9: Experimental ERα binding data for hybrid QSAR models and evaluation. Table S10: Training/test compositions and metrics across 66 ERα hybrid QSAR models. Table S11: Large-scale ERα QSAR inputs from filtered docked PFAS with post-filter descriptors. Table S12: Large-scale ERβ QSAR inputs from filtered docked PFAS with post-filter descriptors. Figure S1: Cross-docking validation of ERα/ERβ native ligands between docked and crystallographic poses, supporting the docking protocol.
