# Supplementary material for: Molecular Determinants of Per- and Polyfluoroalkyl Substances Binding to Estrogen Receptors
Source: Toxics. 2025 Oct 22;13(11):903. doi: 10.3390/toxics13110903 (PMC12655904; doi:10.3390/toxics13110903)
Supplement: Supplementary file 1 [file toxics-13-00903-s001.zip › Supplementary Tables and Figures/Supplementary Tables and Figures.pdf]

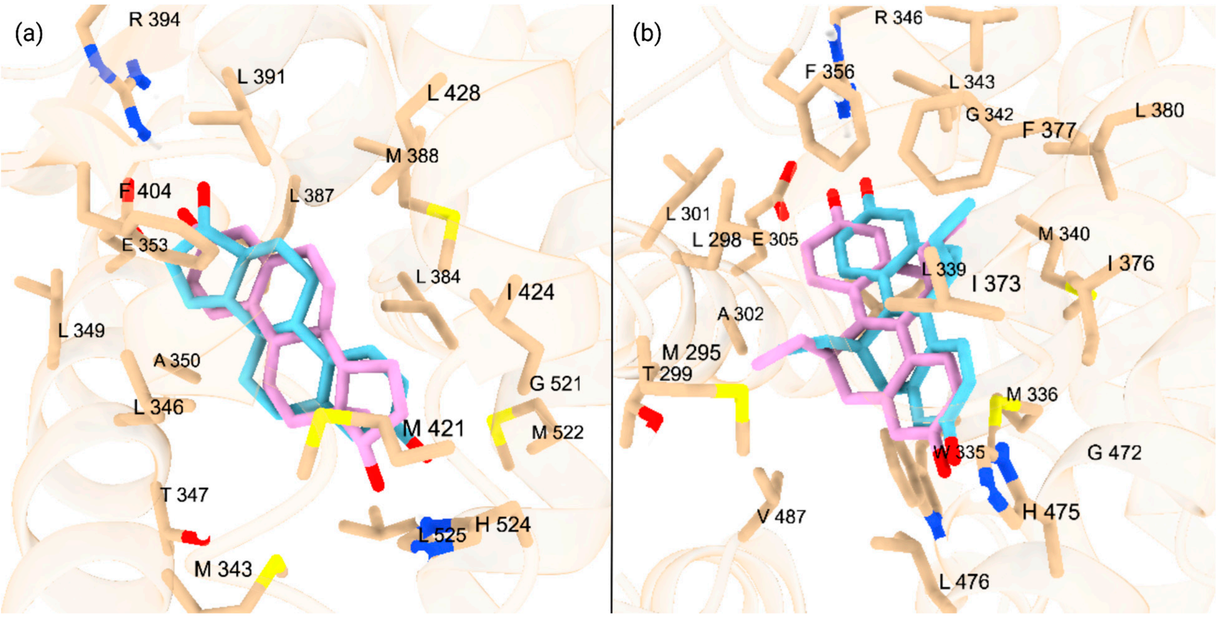


**Supplementary Figure S1 (Figure S1)**: **(a)** Superimposition of docked endogenous ligand, estradiol (pink ligand), over the experimentally observed position of estradiol (blue ligand) at the orthosteric site of ERα **(b)** Superimposition of docked endogenous ligand, (R,R)-5,11-CIS-DIETHYL-5,6,11,12-TETRAHYDROCHRYSENE-2,8-DIOL (pink ligand), over the experimentally observed position of (R,R)-5,11-CIS-DIETHYL-5,6,11,12-TETRAHYDROCHRYSENE-2,8-DIOL (blue ligand) at the orthosteric site of ERβ

**Supplementary Table S6 (Table S6):** List of descriptors used in the generation of the QSPR/QSAR Models and their definitions

| Descriptor | Units | Definition |
|---|---|---|
| **Average Mass** | g/mol | The sum of the atomic weights of all atoms in a molecule |
| **Highest Occupied Molecular Orbital Energy (HOMO)** | eV | The energy of the highest energy molecular orbital that is occupied by electrons |
| **Lowest Unoccupied Molecular Orbital Energy (LUMO)** | eV | The energy of the lowest energy molecular orbital that is unoccupied by electrons |

| | | |
|---|---|---|
| **Number of Freely Rotating Bonds** | N/A | The number of non-terminal single bonds between non-hydrogen atoms that are not part of a ring and are not constrained by conjugation or branching |
| **Number of Hydrogen Bond Acceptors** | N/A | The count of hydrogen bond acceptor atoms in a molecule |
| **Number of Hydrogen Bond Donors** | N/A | The count of hydrogen bond donor atoms in a molecule |
| **LogD (pH 7.4)** | N/A | The logarithm of the distribution coefficient (D) of a compound between n-octanol and water, taking into account both ionized and non-ionized forms at pH 7.4 |
| **Density** | $g/cm^3$ | The mass of a substance per unit volume |
| **Polar Surface Area** | $Å^2$ | The surface area of a molecule occupied by polar atoms |
| **Surface Tension** | dyne/cm | The energy required to increase the surface area of a liquid by a unit amount |
| **F+ Max** | N/A | The maximum electrophilic Fukui index out of all the atoms in a given molecule |

**Supplementary Table S7 (Table S7):** A frequency list of the different classes of PFAS in the commonly exposed dataset. Categories were defined using the EPA classifications.

| Full Category Name | Abbreviation | Count |
|---|---|---|
| N-ethyl perfluoroalkane sulfonamide | EtFASA | 5 |
| N-ethyl perfluoroalkane sulfonamidoethyl acrylate/methacrylate | EtFASAC | 3 |
| Perfluoroalkane sulfonamide | FASA | 1 |
| Fluorotelomer acrylates/alkylacrylate | FTAC | 8 |
| Fluorotelomer iodide | FTI | 4 |
| Fluorotelomer alcohol | FTOH | 6 |
| N-methyl perfluoroalkane sulfonamide | MeFASA | 3 |
| N-methyl perfluoroalkane sulfonamidoethyl acrylate/methacrylate | MeFASAC | 4 |
| Does not fit in ITRC category | NA | 4 |
| Perfluoroalkanoyl fluoride | PAF | 1 |

| | | |
|---|---|---|
| Polyfluoroalkyl phosphate monoester | PAP | 1 |
| Perfluoroalkane sulfonyl fluoride | PASF | 2 |
| Perfluoroalkyl iodide | PFAI | 1 |
| Perfluorocarboxylic acid | PFCA | 9 |
| Perfluoroalkyl Ether Carboxylic Acid | PFECA | 1 |
| Perfluorophosphonic acid | PFPA | 1 |
| Semifluorinated N-alkene | SFene | 1 |
